# Electrophoretic Light Scattering and Electrochemical Impedance Spectroscopy Studies of Lipid Bilayers Modified by Cinnamic Acid and Its Hydroxyl Derivatives

**DOI:** 10.3390/membranes10110343

**Published:** 2020-11-15

**Authors:** Monika Naumowicz, Marcin Zając, Magdalena Kusaczuk, Miroslav Gál, Joanna Kotyńska

**Affiliations:** 1 Department of Physical Chemistry, Faculty of Chemistry, University of Bialystok, K. Ciolkowskiego 1K, 15-245 Bialystok, Poland; joannak@uwb.edu.pl; 2 Doctoral School of Exact and Natural Sciences, University of Bialystok, K. Ciolkowskiego 1K, 15-245 Bialystok, Poland; m.zajac@uwb.edu.pl; 3 Department of Pharmaceutical Biochemistry, Medical University of Bialystok, Mickiewicza 2A, 15-222 Bialystok, Poland; mkusaczuk@wp.pl; 4 Department of Inorganic Technology, Faculty of Chemical and Food Technology, Slovak University of Technology, Radlinského 9, 812 37 Bratislava, Slovakia; miroslav.gal@stuba.sk

**Keywords:** cinnamic acid, *p*-coumaric acid, ferulic acid, phenolic compound, electrophoretic light scattering, electrochemical impedance spectroscopy, phospholipid bilayers, liposomes, drug-membrane interaction, membrane biophysical study

## Abstract

Pharmacological efficiency of active compounds is largely determined by their membrane permeability. Thus, identification of drug-membrane interactions seems to be a crucial element determining drug-like properties of chemical agents. Yet, knowledge of this issue is still lacking. Since chemoprevention based on natural compounds such as cinnamic acid (CinA), *p*-coumaric acid (*p*-CoA) and ferulic (FA) is becoming a strong trend in modern oncopharmacology, determination of physicochemical properties of these anticancer compounds is highly important. Here, electrophoretic light scattering and impedance spectroscopy were applied to study the effects of these phenolic acids on electrical properties of bilayers formed from 1,2-dioleoyl-sn-glycero-3-phosphocholine (DOPC), 1,2-diacyl-sn-glycero-3-phospho-l-serine (PS) or DOPC-PS mixture. After phenolic acid treatment, the negative charge of membranes increased in alkaline pH solutions, but not in acidic ones. The impedance data showed elevated values of both the electrical capacitance and the electrical resistance. We concluded that at acidic pH all tested compounds were able to solubilize into the membrane and permeate it. At neutral and alkaline pH, the CinA could be partially inserted into the bilayers, whereas *p*-CoA and FA could be anchored at the bilayer surface. Our results indicate that the electrochemical methods might be crucial for predicting pharmacological activity and bioavailability of phenolic acids.

## 1. Introduction

A growing body of evidence indicates dietary polyphenols to be efficient antioxidants counteracting negative effects of oxidative stress accompanying many diseases such as cardiovascular diseases, inflammation, and also cancer [1,2,3,4,5,6]. In line with previous reports, many of these compounds may act as chemopreventive or even chemotherapeutic agents [5,7,8,9]. Specifically to malignant cells, cinnamic acid (CinA) and its hydroxy derivatives have been shown to exhibit both antioxidant a antineoplastic activity, with their cytostatic effect being largely related to their structural characteristics [10,11,12,13]. However, the mode of action of CinA and its derivatives is still incompletely determined, but may involve scavenging of reactive oxygen species, modulation of gene expression, activation of xenobiotics metabolism-related enzymes, and regulation of signal transduction pathways essential for tumor cell growth and progression [14].

Despite their natural origin and many promising biological effects, bioavailability of hydroxycinnamic acids presents certain limitations. Although working well in aqueous media, their hydrophilic nature is usually a restriction for lipophilic system protection [15]. Lipophilicity is a major physicochemical property of chemical substances, which affects their biological activities. It is known to be important for describing both pharmacodynamic and pharmacokinetic aspects of drug action. In biological systems, lipophilicity largely determines key properties of potential pharmacological agents, such as solubility of substances in biological fluids, penetration through the biological membranes, rate of absorption, affinity to plasma and tissue proteins, and distribution into the specific body compartments [16]. Lipophilicity is commonly expressed by the logarithm of n-octanol/water partition coefficient (log*P*) for ionizable compounds of a neutral form of compounds. Initially, log*P* was considered important in drug and pesticide discovery and design, but now it is an essential characteristic of all chemicals. This is because log*P* largely determines chemicals fate both inside a living organism and in the environment. Log*P* values are typically between −3 (very hydrophilic) and +10 (extremely hydrophobic) [17]. For ionizable forms of compounds, the distribution coefficient (log*D*) at a specific pH is also often used. As opposed to log*P*, which is only valid for a single electrical species, log*D* represents the pH-dependent mixture of all electrical species occurring at given pH [18].

Based on the observation that most medication drugs are relatively small and lipophilic molecules, Lipinski et al. formulated “the rule of five” (Ro5) [19]. This is a rule of thumb in determining if a pharmacologically/biologically active chemical compound, a candidate for a drug, would be potentially bioavailable via oral administration in humans [20]. According to Ro5, chemicals are less prone to adsorb on the cell membrane and more likely to permeate the bilayer when their calculated n-octanol/water partition coefficient (*c*log*P*) ≥ 5, they have ≥10 H-bond acceptors, ≥5 H-bond donors, and their molecular weight (*M*_WT_) exceeds 500 g/mol [19]. Because each threshold is a multiple of 5, the rule was called Ro5. Molecules not complying with more than one of these rules may have problems with bioavailability [21].

To obtain lipophilicity parameters of significant pharmacokinetic and pharmacodynamic relevance, partition coefficients between (mostly but not exclusively) artificial membranes and water have been tested recently [22]. Monolayers and bilayers have been used for several decades as models of biomembranes to study solute/biological membranes interactions. A variety of lipids and their mixtures may serve as components to prepare both types of these membrane models. Nevertheless, phospholipids tend to be the most widely exploited as they are easily reproducible and have well standardized systems [23]. These lipids are either negatively charged or zwitterionic (electrically neutral due to an equal number of positive and negative charges). Phospholipids are amphipathic and occur naturally in all living organisms as the major components of cell membranes. Most biological membranes are characterized by the asymmetrical distribution of phospholipids within the inner and outer leaflets. The inner leaflet of the bilayer mainly consists of negatively charged lipids, such as phosphatidylserine (PS), while electrically neutral lipids, such as phosphatidylcholine (PC) and phosphatidylethanolamine (PE), are mostly located in the outer layer [24]. At physiological pH, negative charge of the outer leaflet lipids is due to low values of acid dissociation constant (p*K*_a_) of the phosphate moieties of the lipid head group [25].

Our understanding of the properties, function and structure of biological membranes has benefitted a lot from the experimental and theoretical studies of the electrical properties of lipid bilayers. Therefore, evaluation of the parameters such as surface charge density (σ) of the membrane is critical for determination of the electrostatic interactions between membranes and their surrounding solutes. The surface charge of a lipid membrane, which may change with pH, depends also on the lipids present in the outer layer and can be quantified by zeta-potential measurements using electrochemical light scattering (ELS) technique. Zeta potential depends on a lots of parameters, including temperature, pH, conductivity (ionic strength), and solvent (viscosity). Small changes in any of these parameters can potentially dramatically affect the zeta potential values [26].

In order to best mimic electrochemical conditions observed in natural membranes, model systems of artificial bilayers ought to possess similar values of capacitance (*C*_m_) and resistance (*R*_m_) to those observed in biological membranes. In this respect, the parallel plate capacitor model may serve as an estimator of the lipid bilayer capacitance. Assuming that typical hydrophobic thickness of a membrane equals 4 nm and a relative permittivity of ranges from 2 to 4, the capacitance of the bilayer is supposed to be 0.5–1.0 μF/cm^2^. In fact, *C*_m_ values placing within this range have been already confirmed in experimental research [27,28,29]. Typical values of bilayer resistance are in the range 10^4^−10^7^ Ω cm^2^ [30,31]. The measurement of lipid membrane resistance may sometimes be difficult and unreproducible. These variations are most likely a result of a leakage at the bilayer support and/or trapped emulsified droplets [32]. Luckily, membrane capacitance and resistance can be easily measured by electrochemical impedance spectroscopy (EIS). EIS is a technique that enables to extract the information not only about the bulk phase of tested material (e.g., dielectric constant and conductivity) but also about their inner and outer interfaces (e.g., interfacial region capacitance and derived quantities) [33].

**Figure 1:**
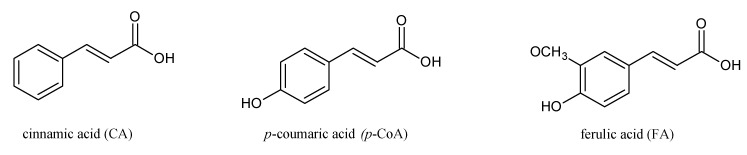
Chemical structures of compounds under study.

Herein, we report on the modulation of the electrical properties of model cell membranes by cinnamic acid and its two naturally occurring hydroxy derivatives: *p*-coumaric acid (*p*-CoA) and ferulic acid (FA). The selected phenolic acids are widely distributed in plants, fungi and algae and recognized as privileged structures for the development of bioactive compounds with therapeutic potential. These compounds are structurally related (Figure 1) and a correlation between their structures and behavior in the surrounding solution seems to warrant further investigations. Although a considerable amount of data concerning hydroxycinnamic acids interactions with membranes exists, still little is known about the electrical properties of bilayer lipid membranes modified by CinA, *p*-CoA or FA. Therefore, this paper is focused on the effect of cinnamic acid and its derivatives on the resistance, capacitance, and the surface charge density of model membranes (spherical lipid bilayers and liposomes).

We previously described changes in the electrical parameters caused by e.g., membrane compositions in lipid–lipid [34], lipid–sterol [35], lipid–fatty acid [36], or lipid-carotenoid membranes [37]. The investigations reported in this paper are a continuation of the studies on the interaction of model membranes of increasing complexity with naturally occurring phenolic compounds. The bilayers were formed from 1,2-dioleoyl-sn-glycero-3-phosphocholine (DOPC), 1,2-diacyl-sn-glycero-3-phospho-l-serine (PS) or from DOPC-PS mixture in concentrations of 9:1 and 8:2 mol%, respectively, which corresponds to PS content in the human cerebral cortex [38]. Next, phospholipid membranes were modified by CinA, *p*-CoA or FA in the concentrations determined on the basis of MTT analysis shown in previous studies on human glioblastoma cell lines. Afterwards, we analyzed the influence of the pH of the electrolyte solution and the composition of membranes on their surface charge to better describe interactions in model membranes modified by the phenolic acids. We also analyzed if CinA, *p*-CoA and FA were capable of altering the electrical resistance and capacitance of the bilayers. The results of our research should give useful indications in understanding the role of chemical structure of hydroxycinnamic acids in determining their interactions with the microenvironment of model lipid bilayer and, thereby, allow us to speculate about the in vivo bioavailability of the investigated compounds.

## 2. Materials and Methods

### 2.1. Reagents

Compounds: trans-cinnamic acid (≥98.0%), *p*-coumaric acid (≥98%), trans-ferulic acid (99%), 1,2-dioleoyl-sn-glycero-3-phosphocholine (>99%) and 1,2-diacyl-sn-glycero-3-phospho-l-serine (≥97%) were obtained from Sigma-Aldrich (St. Louis, MO, USA).

The remaining reagents had the highest degree of purity available on the market and only freshly prepared solutions were used. The electrolyte solutions (155 mM/L NaCl) were prepared using deionized water purified to a resistance of 18.2 MΩ (HLP 5UV System, Hydrolab, Hach Company, Loveland, CO, USA) and filtered using a 0.2-μm membrane filter to eliminate any impurities. All glassware and equipment were cleaned with 18.2 MΩ cm of ultrapure water.

All experiments were conducted at a mean room temperature of 20 ± 2 °C.

### 2.2. Methods

#### 2.2.1. Preparation of Liposomes

Liposomes were prepared by the thin film hydration method to obtain small unilamellar vesicles (SUVs) [39]. Briefly, DOPC, PS, CinA, *p*-CoA or FA were dissolved in chloroform (anhydrous, ≥99%). Lipid alone and phenolic acids solutions were transferred into a glass tube in amounts suitable to obtain the appropriate concentrations of phenolic acids. The concentrations were chosen on the basis of the results of MTT assay performed on human glioblastoma cell lines subjected to CinA, *p*-CoA or FA treatment for 24 and 48 h. Regarding the IC_50_ values and in order to choose the sublethal doses of phenolic acids, the concentrations of 1.0 and 5.0 mmol/dm^3^ have been selected [10,40,41]. A thin lipid film was obtained after the organic solvent evaporation under an argon gas stream to remove any organic solvent residue. The dried lipid film was hydrated with an electrolyte solution (155 mM/L NaCl). Liposomes were formed by sonicating the suspension using the ultrasound generator (UD 20, Techpan, Warsaw, Poland). Sonication was repeated five times, 90 s each. Since heat is released during the process, cooling the suspension is mandatory. It was performed with an ice bath (a container with a mixture of ice and dry sodium chloride). The samples consisted of plain liposomes (10 mg of: DOPC, PS, 9:1 mol% DOPC:PS or 8:2 mol% DOPC:PS), as well as liposomes containing chosen phenolic acid. The liposomes were directly examined in the ELS apparatus.

#### 2.2.2. Preparation of Spherical Bilayers

The stock solutions for bilayers formation were composed of 20 mg cm^−3^ of DOPC or DOPC-PS mixture in concentrations of 9:1 and 8:2 mol%, respectively. Chloroform phospholipids alone and phenolic acids solutions were transferred into a glass tube in amounts suitable to obtain the same concentration of CinA, *p*-CoA or FA as in liposome forming solutions. After mixing the compounds, the solvent was removed under a nitrogen flow, and the resulting dry residues were dissolved in a n-hexadecane-n-butanol mixture (10:1 by volume). All the bilayer-forming solutions were stored in darkness at a temperature of <4 °C in a refrigerator for at least 3 days before examination. To minimize the oxidation of lipids, the vessels containing final samples were filled with nitrogen.

Bilayers were prepared by the method of squeezing the solution, which enables creating spherical bilayers dividing two aqueous solutions. They were formed at the Teflon cap constituting a part of the measuring vessel. During membranes creation, the solvent mixture was removed from the lipid phase, resulting in membranes with the same composition as in stock solutions. Bilayers were monitored both electrically and optically during the entire process of formation. Measurements were initiated 10–15 min after the membranes turned completely black. Membrane images were taken by color CCD camera using the WinFast PVR program. The bilayer areas were calculated from the photographs, taking into consideration the spherical nature of the surface and employing the Makroaufmassprogram program [42]. The area of the spherical membranes was about 6 × 10^−2^ cm^2^. More details regarding the procedure for the membrane formation are given in our previous works [31,36,37].

#### 2.2.3. Electrophoretic Light Scattering Measurements

Microelectrophoretic mobility of the liposomes was obtained by performing micro-electrophoretic assessments on samples using ELS technique. The measurements were performed by Zetasizer Nano ZS apparatus (Malvern Instruments, Malvern, United Kingdom). The xperiment was carried out as a function of pH using a WTW InoLab pH 720 laboratory meter (WTW, Weinheim, Germany). The samples were suspended in 155 mM/L NaCl solution and titrated to the desired pH (range 2–10, every ± 0.3 units) with HCl or NaOH. Six measurements were made (each covering 100–200 series for a duration of 5 s) for each pH value per sample. Experiments were performed three times with similar results.

The surface charge density *δ* depends on electrophoretic mobility as described by the following equation [43]:

(1) $$\delta =\frac{\eta \cdot u}{d}$$

where *η* is the viscosity of the solution, *u* is the electrophoretic mobility, and *d* is called the diffuse layer thickness.

The diffuse layer thickness is defined as [44]:

(2) $$d=\sqrt{\frac{\varepsilon \cdot \varepsilon_{o}\cdot R\cdot T}{2\cdot F^{2}\cdot I}}$$

where: *R* represents the gas constant, *T*—the temperature, *F*—the Faraday constant, *I*—the ionic strength of 0.9% NaCl, and *ε* and *ε*_0_ refer to the permeability of the electric medium.

#### 2.2.4. Liposome Size Determination

Dynamic light scattering (DLS) method was used to determine the size of the liposomes. The diameter of the particles was evaluated from the intensity of the dispersed light, which is the standard parameter measured, by the Zetasizer Nano ZS software. A helium–neon (He–Ne) ion laser at 633 nm wave length was used as the incident beam.

#### 2.2.5. Electrochemical Impedance Measurements

Electrochemical impedance spectroscopy was conducted with an Autolab potentiostat (Model PGSTAT302N, Metrohm, Poland) coupled with an FRA2 module. A four-probe cell with two Ag/AgCl reference electrodes was employed for sensing and two platinum electrodes were used to carry the current for the measurements. The cell was presented in [37]. EIS data were registered in the frequency range of 0.1–100,000 Hz. A sinusoidal voltage excitation of 4 mV versus open circuit potential was applied. No direct current was used.

**Scheme 1:**
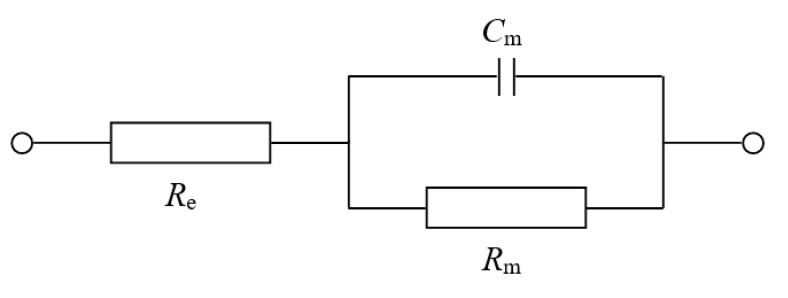
The equivalent circuit model used to fit EIS data.

Analysis of the data obtained during EIS measurements was performed using NOVA software (v. 1.10) with the fitting model shown in Scheme 1. This electrical circuit was described previously for the lipid bilayer unmodified by channels formers or carriers [45]. The circuit consists of the solution resistance (*R*_e_) in series with a membrane resistance (*R*_m_) and a membrane capacitance (*C*_m_), which are in parallel and correspond with the electric and dielectric properties of the bilayer. The values of the *R*_m_ and *C*_m_ were normalized to the bilayer area. Each value represents the average of 6–10 independent measurements.

## 3. Results

In order to get a deeper insight into the interactions between CinA, *p*-CoA or FA and bilayer lipid membranes, a systematic series of experiments was carried out using two representative concentrations of each phenolic acid. From the results of previous tests carried out on human glioblastoma cell lines [10,40,41], 1 and 5 mM/L concentrations have been selected. Firstly, the surface charge density of phospholipid liposomal membranes (DOPC, PS, DOPC/PS 9:1 and DOPC/PS 8:2), plain and modified by phenolic acids, was calculated based on the electrophoretic light scattering (ELS) technique. Subsequently, the capacitance and resistance values of DOPC, DOPC/PS 9:1 or DOPC/PS 8:2 spherical bilayers (plain and phenolic acid-modified) were obtained on the basis of the impedance data.

### 3.1. The Effect of Cinnamic Acid and Its Derivatives on Surface Charge Densities of Liposomal Membranes

To get insight into the surface charge density-modulatory properties of CinA, *p*-CoA and FA, the ELS technique has been engaged. The measurements were performed as a function of H^+^ concentration. In order to receive the values of pH-dependent electrophoretic mobility, liposomes were suspended in 155 mM/L NaCl solution, which was further titrated to the adequate pH with concentrated NaOH or HCl. Surface charge density values were calculated on the basis of electrophoretic mobility in accordance to Equation (1) provided in section “Materials and Methods”. Representative plots from at least three independent experiments are shown.

**Figure 2:**
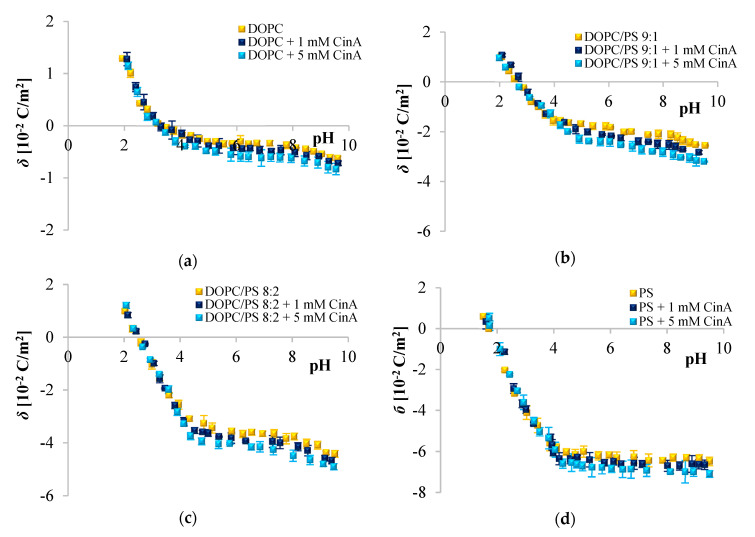
pH dependence of surface charge density of (**a**) DOPC, (**b**) DOPC/PS 9:1, (**c**) DOPC/PS 8:2, and (**d**) PS liposomal membranes as a function of (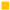) 0, (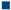) 1, and (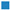) 5 mM/L of cinnamic acid concentration. The results represent mean values from three independent experiments run in triplicate.

Surface charge density dependence on pH of plain and CinA-modified DOPC, DOPC/PS 9:1, DOPC/PS 8:2 and PS liposomal membrane occurred to be similarly shaped (Figure 2). An increase in positive surface charge density together with the decrease in pH values was noticed, however, only to a certain point. Conversely, along with the increasing pH, the negative charge of the membranes incremented until the plateau was reached. The visible decrease in the surface charge of PC-containing membranes at pH ~ 8.0 was possibly caused by the destruction of the membrane structure at such a high pH, which is in line with earlier reported data [7]. Our results demonstrated that in low pH, any visible alterations in values of the surface charge density were detected for all tested liposomal membranes treated with CinA. Nevertheless, at high pH values, the presence of CinA resulted in visible changes in surface charge densities of the aforementioned membranes (the negative charge of the membranes increased) compared to data obtained for plain phospholipid liposomal membranes. Those changes were apparently dose-dependent.

**Figure 3:**
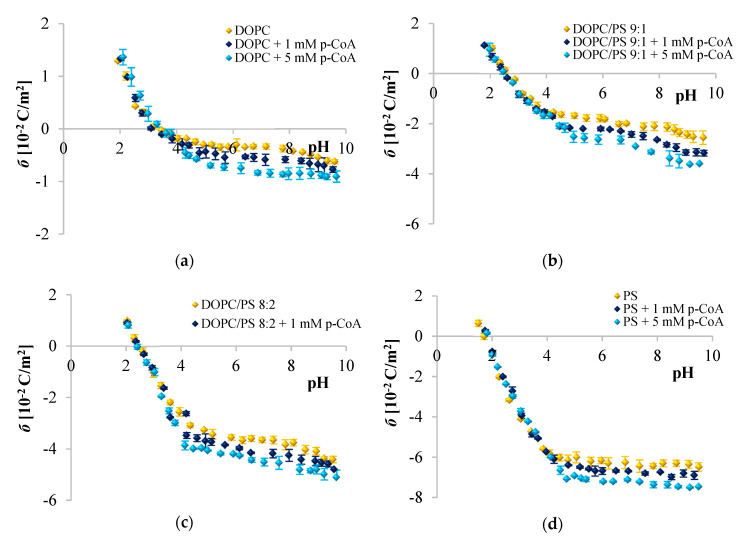
pH dependence of surface charge density of (**a**) DOPC, (**b**) DOPC/PS 9:1, (**c**) DOPC/PS 8:2, and (**d**) PS liposomal membranes as a function of (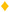) 0, (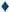) 1, and (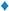) and 5 mM/L of *p*-coumaric acid concentration. The results represent mean values from three independent experiments run in triplicate.

The liposomes treated with *p*-coumaric acid were examined analogously (Figure 3). It was found that in acidic solution, the presence of *p*-CoA did not affect the *σ* values. However, in alkaline solutions, an increase in the negative charge of *p*-CoA-treated membranes in comparison to plain phospholipid liposomal membranes was observed.

**Figure 4:**
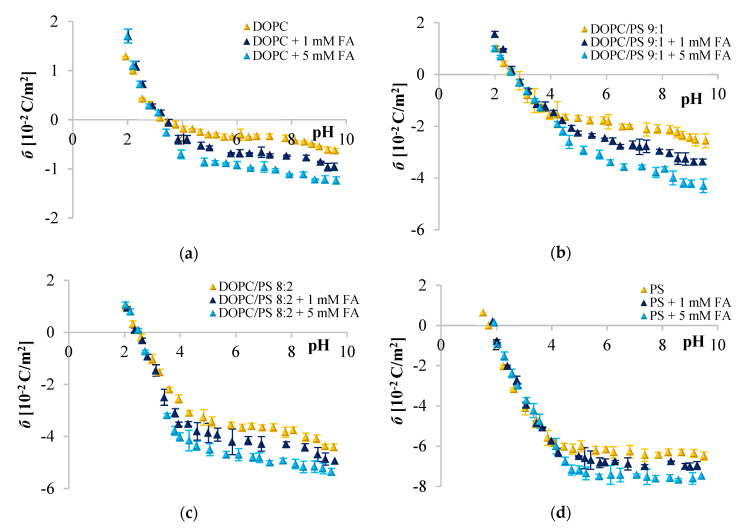
pH dependence of surface charge density of (**a**) DOPC, (**b**) DOPC/PS 9:1, (**c**) DOPC/PS 8:2, and (**d**) PS liposomal membranes as a function of (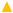) 0, (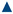) 1, and (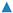) 5 mM/L of ferulic acid concentration. The results represent mean values from three independent experiments run in triplicate.

The surface charge densities of the DOPC, DOPC/PS 9:1, DOPC/PS 8:2, and PS liposomal membrane, plain and modified with ferulic acid, are shown as a function of pH in Figure 4. Again, the presence of the phenolic acid did not affect the *σ* values at low pH, but caused an increase in the negative *σ* values at higher pH ranges.

Considering the ELS data presented in Figure 2, Figure 3 and Figure 4 and comparing membranes with the same phospholipid composition, it can be observed that the smallest changes in the surface charge density values were obtained in the presence of the most hydrophobic of all, cinnamic acid, whereas the most prominent alterations were noticed in the presence of the most hydrophilic compound, ferulic acid.

Finally, the results collected in Figure 2, Figure 3 and Figure 4 illustrate that the presence of CinA, *p-*CoA or FA did not change the value of the isoelectric point of all analyzed phospholipid membranes. Since the isoelectric point of the membrane is one of the most important parameters describing its variable-charge surfaces, it is important to point out that it has shifted towards lower pH values for DOPC as compared to PS (decrease from pH ∼3.5 to pH ∼1.7, respectively). This is coherent with other investigations demonstrating that the isoelectric point of PC lipids fits into the pH values ranging between 3.2 and 4.0 [41,46,47], while reaching 1.4–1.7 values for PS lipids [41,46].

### 3.2. The Effect of Cinnamic Acid and Its Derivatives on the Size of the Phospholipid Liposomes

The effect of the analyzed phenolic acids on the size of the phospholipid liposome systems is presented in Table 1, Table 2 and Table 3. The modification of liposome membranes with CinA, FA and p-CoA resulted in variations in the size of the examined systems. Notably, the diameter of the liposomes was also found to be influenced by the applied doses of the phenolic acids (1 and 5 mM/L). These changes are observed in both acidic (pH ~ 2) as well as alkaline (pH ~ 9) pH.

Considering the data collected in Table 1, it can be observed that for plain DOPC liposomes as well as for DOPC liposomes treated with cinnamic acid, sizes of the liposomes increase along with increasing pH of the medium. The same tendency was observed for membranes treated with *p*-coumaric and ferulic acids (Table 2 and Table 3). However, the opposite trend was noted for negatively charged liposomes (DOPC/PS 9:1, DOPC/PS 8:2, PS). Here, sizes of both plain and modified liposomes decreased together with increasing pH. In acidic pH, the diameter of phospholipid liposomes modified with cinnamic acid did not vary significantly from the diameter of liposomes modified with p-coumaric and ferulic acids. In alkaline pH, phospholipid liposomes treated with ferulic acid exhibited the largest sizes, whereas those treated with cinnamic acid were the smallest.

**Figure 5:**
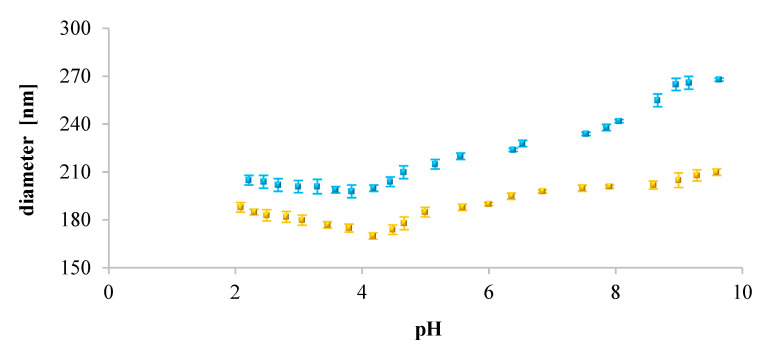
Typical pH dependence of the diameter of the liposomes formed by DOPC (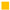) and DOPC modified with 5 mM/L ferulic acid (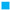). The results represent mean values from three independent experiments run in triplicate.

Figure 5 presents typical pH dependence of the diameter of the liposomes created with pure DOPC and DOPC modified with one of the analyzed phenolic acids, FA. Of note, in pH > 4, the sizes of plain DOPC liposomes and DOPC modified with ferulic acid increased with increasing pH of the medium, whereas in pH ~ 4 (isolectric point of the system), the diameter of analyzed liposomes was the lowest.

### 3.3. The Effect of Cinnamic Acid and Its Derivatives on Capacitance and Resistance of Spherical Bilayers

Electrochemical impedance spectroscopy was utilized to quantify the effect of the examined phenolic acids on the membrane resistance (reciprocal of conductance) and capacitance. This method seems to be non-destructive and presents high sensitivity to feasible drug-induced modulation of bilayer thickness or packing [48]. As such, EIS was used over a wide range of frequencies from 10^−1^ Hz to 10^4^ Hz under small amplitude. The 155 mM/L NaCl electrolyte solution was used to register impedance spectra.

Two different phospholipids i.e., DOPC and PS, were used to prepare one- or two-component bilayers. Unfortunately, bilayers composed only from PS did not have the ability to form sufficiently stable membranes for the impedance measurements. CinA, *p*-CoA or FA was introduced to the phospholipid model membrane solution at concentrations of 1 and 5 mM/L. In order to extract the *R*_m_ and *C*_m_ values from the impedance spectra, the EIS data were fitted to the equivalent circuit model shown in Scheme 1 presented in section “Materials and Methods”. In the following analyses, the values of the impedance parameters referred to the bilayer surface area unit. At least six spherical lipid bilayers were tested for consistency. For quantitative data, average values with standard deviations are reported.

**Figure 6:**
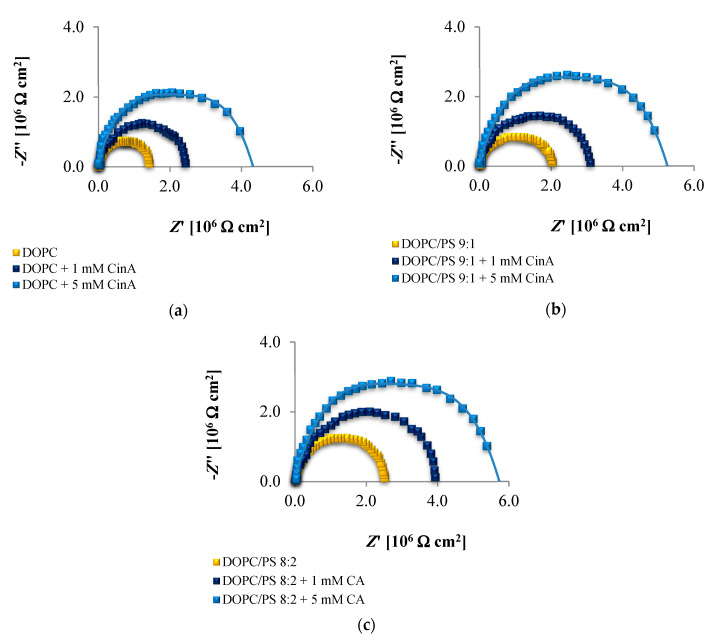
Representative impedance spectra for (**a**) DOPC, (**b**) DOPC/PS 9:1, and (**c**) DOPC/PS 8:2 spherical bilayers as a function of (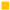) 0, (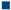) 1, and (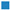) 5 mM/L of cinnamic acid concentration. Experimental data points are indicated with markers; model fits are shown with lines.

Figure 6 displays the impedance response in the form of Nyquist plots of different spherical bilayers, with varied concentrations of CinA. Notably, the plot registered for each bilayer contains a capacitive arc across the entire frequency range. The diameter of the arc corresponds to the membrane resistance. The incorporation of the CinA into the DOPC, DOPC/PS 9:1 or DOPC/PS 8:2 membrane resulted in an increase in the *R*_m_ values (compared to the values obtained for bilayers with the same lipid composition but without phenolic acid), confirming that this compound is active at the tested concentrations.

Table 4 provides the values of the electrochemical elements obtained by fitting the electrochemical impedance spectra. The typical value of the electrolyte resistance was equal to (5.57 ± 0.20) × 10^3^ Ω and was considered irrelevant to the bilayers characteristics. Therefore, this parameter was omitted in the curve fitting procedure. The *R*_m_ value of the DOPC bilayer was evaluated prior to the addition of the phenolic acid and found to be (1.472 ± 0.07) × 10^6^ Ω cm^2^, which is consistent with the reported values for other PC known to typically range between 10^4^ and 10^7^ Ω cm^2^ [30,31]. The obtained *C*_m_ value for plain DOPC membrane was (0.617 ± 0.03) μF/cm^2^, which is in line with the capacitance values of PC bilayers calculated based on the chronopotentiometric [27], chronoamperometric [49] or impedance [45] method. As presented in Table 4, the increase in membrane capacitance was also noticed after the addition of CinA to the spherical bilayers.

**Figure 7:**
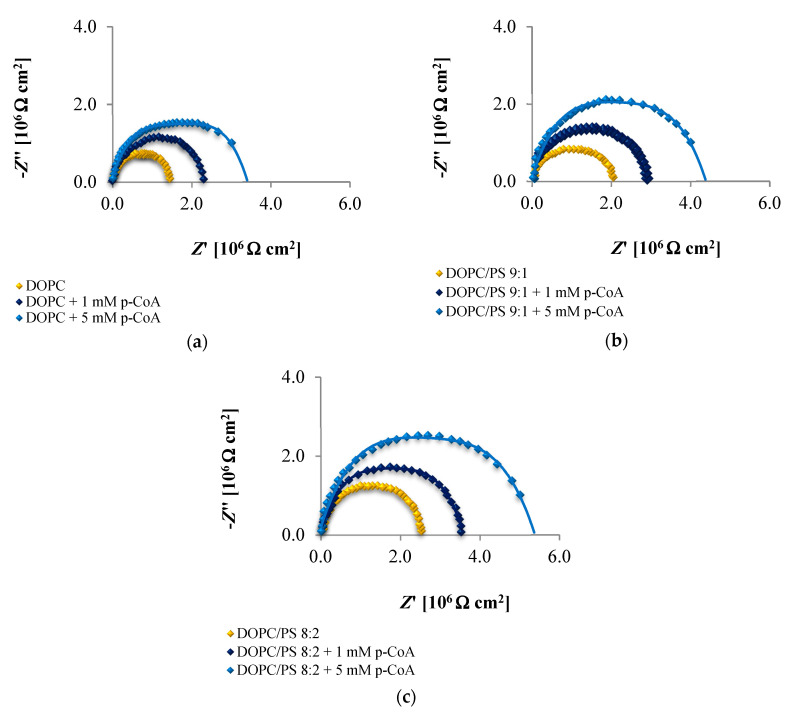
Representative impedance spectra for (**a**) DOPC, (**b**) DOPC/PS 9:1, and (**c**) DOPC/PS 8:2 spherical bilayers as a function of (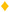) 0, (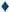) 1, and (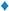) 5 mM/L of *p*-coumaric acid concentration. Experimental data points are indicated with markers; model fits are shown with lines.

Three independent curves obtained for membranes with the same phospholipid composition, presented in Figure 7a–c, clearly show that adding increasing concentrations of *p*-CoA to the DOPC, DOPC/PS 9:1 or DOPC/PS 8:2 bilayers led to an increase in the membrane resistance.

Table 5 summarizes the results concerning the influence of the membrane composition on the capacitance and resistance values. Upon introducing *p*-CoA, an increase in both parameters was detected compared to the plain membranes.

**Figure 8:**
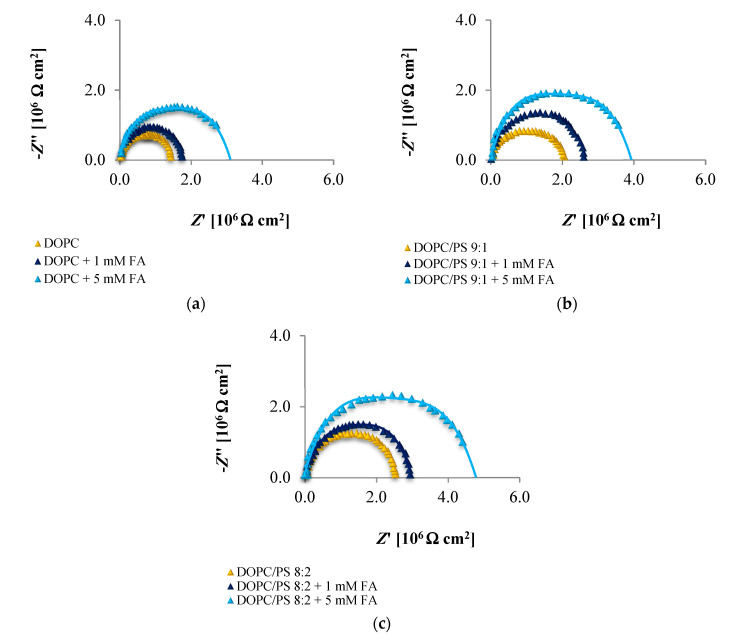
Representative impedance spectra for (**a**) DOPC, (**b**) DOPC/PS 9:1, and (**c**) DOPC/PS 8:2 spherical bilayers a function of (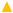) 0, (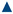) 1, and (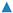) 5 mM/L of ferulic acid concentration. Experimental data points are indicated with markers; model fits are shown with lines.

From the subsequent EIS data presented in Figure 8 and Table 6, it is evident that both the bilayer resistance and capacitance increase in the presence of FA.

Taking into account the data gathered in Figure 6, Figure 7 and Figure 8 and in Table 4, Table 5 and Table 6 and by comparing membranes with the same phospholipid composition, it may be noted that the smallest changes in the capacitance and the biggest changes in the resistance values were observed in the presence of the most hydrophobic of all tested compounds—cinnamic acid. Conversely, the largest alterations in the capacitance and the lowest in the resistance were noticed in the presence of the most hydrophilic one, ferulic acid.

## 4. Discussion

Cinnamic acid and its derivatives have attracted the attention of more and more researchers due to the broad variety of pharmaceutical and biological activities and due to the wide technological or industrial applications [50]. Polyphenols are important primarily because of their antioxidant property, antitumor activity or hypertension-preventing effect. Interactions of these phenolic acids with cell membranes play an essential role in their transport, distribution, selectivity, activity, and toxicity.

Small organic acids such as hydroxycinnamic acids have been postulated to interact with/and penetrate into biological membranes, however, their ability to demonstrate such an interaction with lipid membranes can be greatly influenced by the type of substituents attached to the main structure. Therefore, structurally similar compounds may differently interact with biological membranes, because of the complex relationship (which can be modulated by pH changes) between liposolubility and permeability [51]. The dissociation constant is a very important parameter describing solubility, lipophilicity, and permeability properties of compounds. Phenolic acids in aqueous solutions exhibit protonated and deprotonated forms. Analyzing the values of dissociation constants of the carboxyl and hydroxyl groups of the studied phenolic acids (Table 7), it can be stated that their molecular and monodeprotonated forms prevail within the range of physiological pH values. Moreover, the corresponding dissociation constant values are all similar to each other, which demonstrates that they are not a main factor determining the activity of the hydroxycinnamic acids. However, these data could be useful in studies concerning bioavailability and pharmacokinetics of potential pharmacological agents [52].

The positive values of the calculated log*P* of CinA, *p*-CoA and FA may indicate that these acids are partitioning in the octanol phase. Nevertheless, only lipophilicity of uncharged molecules can be determined by this method, thus it is difficult to apply to these phenolic acids, which at physiological conditions are rather negatively charged. Analysis of Table 7 revealed that there are relevant differences between *c*log*P* and log*D* coefficient partitions for each acid. These findings suggest that the drug distribution between the aqueous solution and the lipid membrane is regulated by the ionization state of the molecule. All three compounds tested here are ionizable molecules, and it is already widely recognized that ionizable drugs partition into the lipid membrane to a high extent thanks to electrostatic interactions and formation of hydrogen bonds with polar groups of the phospholipids [18]. As a matter of fact, several reports inform about different experimental log*D* values from the predicted log*P* values calculated exclusively for the neutral species of drugs [58,59].

Rocher et al. reported the percentage of the undissociated forms of i.e., CinA, *p*-CoA and FA in pulvinar cells of *Mimosa pudica* L. at pH 5.2, which amounted to 70.4%, 58.6%, and 52.7% of the total pool of these acids respectively [18]. Considering the fact that only lipophilic neutral forms are able to cross the plasma membrane by diffusion, this parameter bears important information on this family of carboxylic acids [60].

Regarding the importance of physicochemical properties of drugs, Lipinski developed Ro5 [18]. According to this rule, candidates for efficient drugs should be characterized by log*P* ≤ 5, molecular weight ≤ 500, number of hydrogen bond acceptors (O) ≤ 10, and number of hydrogen bond donors (OH, NH) ≤ 5. All phenolic acids examined herein meet the Lipinski’s rule as demonstrated in Table 7. Thus, CinA, *p*-CoA and FA display favorable drug-like properties, which certainly encourage their further examination in the in vitro and in vivo studies.

Since phenolic compounds has been demonstrated as efficient cytostatic agents against various malignancies, it seems essential to evaluate how these compounds interact with lipid membranes, to get fuller insight into the transportation and anticancer mechanisms of these natural compounds. The affinity of polyphenols to the lipid bilayer is reflected in several electrochemical parameters. The first determinant is the adsorption on the membrane surface mediated by interactions of hydrophilic parts with the polar head groups of the lipids at the water–lipid interface. Second is the absorption dependent on the partitioning of the hydrophobic parts into the nonpolar core of the membrane [61,62]. The mechanism of action of polyphenols is determined by the presence of different substituents in their backbone structure and the pH value of their microenvironment. If the pH of the external medium is low (acidic), phenolic acids are then able to diffuse through the membrane due to their unchanged form [63,64]. The ELS data presented in Figure 2, Figure 3 and Figure 4 clearly show that cinnamic acid and its derivatives do not affect the surface charge density of liposomal membranes at acidic pH. Therefore, it can be assumed that at acidic pH, investigated polyphenols were able to solubilize into the membrane and to permeate it. At neutral and basic pH, the CinA was unable to considerably modify the surface charge of the model membrane due to the lack of a hydroxyl group. Whereas more hydrophilic *p*-CoA and FA remained anchored at the bilayer surface without perturbing the lipidic structure but clearly affecting *σ* values. These findings are in agreement with literature, where the influence of structural characteristics of cinnamic acid and its hydroxyl derivatives on their interaction with model membranes was reported [51,65,66]. Similarly, the penetration of many other compounds e.g., flavonoids [7] or non-steroidal anti-inflammatory drugs [67] in bilayers, depends on the pH of the media.

Given the *p*K_COOH_ values (Table 7), at neutral and basic pH, the carboxyl group of CinA, *p*-CoA and FA is most likely negatively charged, and the negative charge of their molecules is probably oriented towards the positive pole of zwitterionic DOPC headgroup. ELS measurements performed in the presence of two concentrations of phenolic acids corroborate this hypothesis given that the surface charge of the membrane was altered, becoming increasingly negative with the addition of the examined polyphenols. The same tendency was observed for other negatively charged compounds [67]. The incorporation of cinnamic acid resulted in the slightest changes of *σ*, which again suggests interactions of CinA with the interior of the membrane. It is obvious that the effects of different phenolic acids are correlated to their structural characteristics, thereby even the difference in one –OH group can be important, as well as the number of H-bonds they form. CinA can form three H-bonds, with one as a H-bond donor, and two as H-bond acceptors; its partition coefficient was the highest, and it was the most soluble of these phenolic acids in octanol (Table 7). In other words, CinA as a non-polar substance could enter the lipid bilayer easily. Our findings are in line with the literature, where the interaction of model membranes with phenolic acids presenting different structural changes in their molecular backbone is deciphered on the basis of the shift of lipid phase transition temperature [51,61,66,68]. These authors reported that for pH below p*K*_COOH_ values, the protonation of the carboxylic group allows the substance to penetrate the lipid bilayer. Likewise, Castelli et al. employed multilamellar or unilamellar liposomes created from synthetic L-R-dimyristoylphosphatidylcholine to check whether cinnamic acid can dissolve into lipid membranes and penetrate them by migration from the aqueous phase [51]. This process continues until there is a constant molar fraction on the membrane surface, and then progressively inside the other internal bilayers. At the end of this process, the thermotropic behavior is close to that obtained by direct mixing of the biophenol with the lipid component during the liposomal preparation. The same researchers investigating PC liposomes containing *p*-CoA at two different pHs (4 and 7) reported no shift of the calorimetric peaks toward lower values, suggesting that the –OH group influences the ability of this compound to penetrate the membrane [51]. They did not exclude a surface interaction with the lipid layers and stated that the presence of different substituents in the backbone structure of biophenols might influence their incorporation. On the other hand, Ota et al. compared the thermograms obtained for the unilamelar large vesicles of 1,2-dipalmitoyl-sn-glycero-3-phosphocholine in the presence of the *p*-CoA or FA, and noticed that the enthalpy value of the main transition of the phospholipids decreased by 1.46 ± 0.10 kcal/mol in the presence of *p*-CoA, and by 0.45 ± 0.17 kcal/mol with FA. This small but significant decrease in the enthalpy of transition implies that both acids intercalate into the acyl chain region of the bilayer [66].

Analyzing the influence of the polar headgroup of lipids on the penetration capacity of cinnamic acid and its derivatives, we expected that phenolic acids will be less able to penetrate the negatively charged headgroup of PS lipids as compared with DOPC. Surprisingly, we have not noticed such an effect. We speculate that perhaps the acids concentrations used here were not sufficient to cause significant alterations of the membrane surface charge, or it may also be the result of certain limitations of the ELS technique per se. Likewise, Fadel et al. [64] reported that rosmarinic acid, another compound belonging to phenolic acids, evoked a weaker effect in PS than PC membranes.

Furthermore, we analyzed the influence of pH on the size of the liposomes. Our findings showed that for neutral liposomes (DOPC), their diameter increased with increasing pH of the solution. It may suggest that in acidic pH, existing electrostatic repulsive forces dominate over hydrogen bonding affinities between neighboring lipid molecules [69]. This might be a tentative explanation of why we observed smaller sizes of DOPC liposomes. Contrarily, for the negatively charged liposomes (DOPC/PS 9:1, DOPC/PS 8:2, PS), the diameter decreased with increasing pH. As such, at acidic pH, the extent of protonation was higher than this observed in the case of DOPC liposomes. Also, an opposite dependence, the dominance of hydrogen bonding over electrostatic repulsions, was observed. This might be the reason why the negatively charged liposomes showed larger sizes than neutral ones (DOPC). Simultaneously, in alkaline and neutral pH, reduction of the diameter of negatively charged liposomes was observed most probably due to the repulsive forces between phospholipid molecules [70].

The EIS data presented in Figure 6, Figure 7 and Figure 8 and collected in Table 4, Table 5 and Table 6 indicate that the addition of cinnamic acid and its hydroxy derivatives to the DOPC, DOPC/PS 9:1 or DOPC/PS 8:2 membranes caused an increase in their resistance, and thus reduced conductivity. An increase in the *R*_m_ value implies that these phenolic acids exacerbated the ordering and decreased the dynamics of the phospholipid alkyl chains of spherical bilayers. This behavior can be explained in relation to the polarities of the examined molecules, indicating to what levels they can penetrate into the lipid bilayer. CinA, the least polar acid among the tested ones, had the greatest effect on the structure of the membrane lipids (i.e., stabilizing of the structure). *p*-CoA and FA are both more polar than CinA (see Figure 1), therefore, presenting a weaker effect on the membrane resistance. Based on our experimental approach, the following order of the stabilization effect was established: cinnamic acid > *p*-coumaric acid > ferulic acid. Our results are in line with previously reported data where the influence of the phenolic acids on structural properties of a model lipid membrane was investigated by differential scanning calorimetry [51,66,68], fluorescence spectroscopy [66,68], and electron paramagnetic resonance spectroscopy [68].

As opposed to the resistance, alterations in the capacitance seem to be less obvious in Nyquist plots, therefore, no remarkable differences between subsequent EIS measurements can be observed in Figure 6, Figure 7 and Figure 8. It may be stated that the deposition of phenolic acids on the membrane was not accompanied by the appearance of any additional time constant, which was also reported not to happen in the case of protein adsorption [71]. Consequently, the *C*_m_ values fitted from the equivalent circuit model were in alignment with the indications from the Nyquist (Table 4, Table 5 and Table 6), revealing increasing capacitance with increasing phenolic acids concentrations.

Together, the EIS data indicate that in comparison to untreated plain phospholipid bilayer, CinA-stimulated membranes showed a significant and systematic increase in bilayer resistance, which is dependent on increasing the concentration of phenolic acid. These changes were accompanied by moderate elevation of the bilayer capacitance, which can be attributed to a decrease in membrane thickness. This in turn, may indicate the ordering and stabilizing effect of CinA on the phospholipid alkyl chains of bilayers.

According to the EIS response shown in Figure 6, Figure 7 and Figure 8 and data collected in Table 4, Table 5 and Table 6, it is evident that *p*-CoA and FA interact with the bilayer in a different way than CinA. Both of these compounds elicit significant changes in the lipid membrane capacitance, which indicate their adsorption at the bilayer interface. In contrast to CinA, changes in membrane resistance caused by increasing concentrations of *p*-CoA or FA were much smaller. These observations were consistent with the ELS data, suggesting that the CinA associates strongly and penetrates deeply into the lipid membrane. Conversely, *p*-CoA and FA may locate near the phospholipid headgroup, most probably via electrostatic interaction, without perturbing the lipidic structure.

Finally, if we compare the influence of the polar headgroup of lipids on the *R*_m_ and *C*_m_ values of membranes modified with CinA, p-CoA or FA, it seems that phenolic acids are less able to interact with the negatively charged headgroup of PS lipids as compared with DOPC. This result is not surprising, because the data were registered at pH equals 6.59 (pH of 155 mM/L NaCl electrolyte solution), in which all tested compounds bear a negative charge (carboxylate group) that may cause charge repulsion between the two carboxylate functions of the serine in PS. Notably, we failed to identify the differences in the interaction between phenolic acids and DOPC or PS bilayers using zeta potential analysis. This indicates that impedance measurement is a useful and effective technique worth utilizing in physicochemical studies.

## 5. Conclusions

Currently, in the era of lipidomics, the attention of scientists from many fields has been shifted towards looking at biological membranes from a different perspective. Membranes have been demonstrated to determine certain physiological functions of cells and play important roles in several pathologies such as cancer. In this respect, a great deal of attention is now directed into the understanding of the interactions between anticancer drugs and cellular membranes. Thus, evaluation of drug-membrane dependencies can serve as a useful tool in predicting membrane permeability, bioactivity and cytotoxicity of potential antineoplastic agents in modern oncopharmacology. Regarding the importance of such interactions, the role of biomimetic model membranes and biophysical/electrochemical techniques becomes increasingly significant in chemical and pharmacological studies. As such, based on previous in vitro research conducted on glioblastoma cancer cells, we used electrophoretic light scattering and impedance spectroscopy to study the effects of CinA, *p*-CoA and FA on electrical properties of bilayers formed from DOPC, PS or DOPC-PS mixture. We demonstrated that after treatment with phenolic acids, the negative charge of membranes increased in alkaline pH solutions, but not in acidic ones. On the other hand, the data from impedance measurements showed elevated values of either the electrical capacitance and the electrical resistance in treated cells. We therefore concluded that at acidic pH, all tested compounds were able to solubilize into the membrane and permeate it. However, at neutral and alkaline pH, the CinA could be partially inserted into the bilayers, whereas *p*-CoA and FA could be anchored at the bilayer surface. Since intracellular penetration seems to be a key determinant of drug functioning, our results imply that electrochemical methods might be employed for predicting pharmacological activity and bioavailability of phenolic acids in the future.

## Figures and Tables

**Table 1:** Effect of cinnamic acid on the diameter of the liposomes at different pH.

System	Diameter [nm]	
	pH ~ 2	pH ~ 9
DOPC	180.2 ± 3.2	205.5 ± 4.6
+1 mM/L CinA	195.2 ± 4.1	220.2 ± 3.6
+5 mM/L CinA	203.0 ± 2.0	234.2 ± 2.4
DOPC/PS 9:1	257.1 ± 4.2	219.2 ± 4.6
+1 mM/L CinA	253.0 ± 4.2	234.3 ± 3.6
+5 mM/L CinA	259.5 ± 3.0	248.0 ± 2.4
DOPC/PS 8:2	290.2 ± 2.2	240.2 ± 4.5
+1 mM/L CinA	297.0 ± 4.4	262.3 ± 4.6
+5 mM/L CinA	305.2 ± 2.1	277.4 ± 1.4
PS	340.3 ± 3.3	270.0 ± 2.1
+1 mM/L CinA	348.1 ± 4.5	284.1 ± 4.8
+5 mM/L CinA	360.3 ± 2.4	298.0 ± 3.1

**Table 2:** Effect of *p*-coumaric acid on the diameter of the liposomes at different pH.

System	Diameter [nm]	
	pH ~ 2	pH ~ 9
DOPC	180.2 ± 3.2	205.5 ± 4.6
+1 mM/L *p*-CoA	188.4 ± 3.1	227.3 ± 2.5
+5 mM/L *p*-CoA	200.1 ± 4.0	241.2 ± 2.8
DOPC/PS 9:1	257.1 ± 4.2	219.2 ± 4.6
+1 mM/L *p*-CoA	259.2 ± 2.3	240.4 ± 3.0
+5 mM/L *p*-CoA	270.1 ± 2.6	260.1 ± 2.9
DOPC/PS 8:2	290.2 ± 2.2	240.2 ± 4.5
+1 mM/L *p*-CoA	299.0 ± 5.4	273.0 ± 3.4
+5 mM/L *p*-CoA	310.5 ± 3.5	290.3 ± 2.4
PS	340.3 ± 3.3	270.0 ± 2.1
+1 mM/L *p*-CoA	351.1 ± 3.0	290.2 ± 4.0
+5 mM/L *p*-CoA	360.1 ± 2.7	315.4 ± 3.1

**Table 3:** Effect of ferulic acid on the diameter of the liposomes at different pH.

System	Diameter [nm]	
	pH ~ 2	pH ~ 9
DOPC	180.2 ± 3.2	205.5 ± 4.6
+1 mM/L FA	189.1 ± 4.0	249.2 ± 3.1
+5 mM/L FA	201.4 ± 4.5	265.2 ± 3.8
DOPC/PS 9:1	257.1 ± 4.2	219.2 ± 4.6
+1 mM/L FA	268.3 ± 5.3	248.6 ± 3.0
+5 mM/L FA	280.4 ± 5.6	260.4 ± 2.9
DOPC/PS 8:2	290.2 ± 2.2	240.2 ± 4.5
+1 mM/L FA	298.4 ± 3.4	276.5 ± 3.4
+5 mM/L FA	319.6 ± 3.5	301.7 ± 2.4
PS	340.3 ± 3.3	270.0 ± 2.1
+1 mM/L FA	347.5 ± 3.5	299.0 ± 4.4
+5 mM/L FA	359.1 ± 2.7	320.1 ± 3.4

**Table 4:** Effect of cinnamic acid on the resistance and capacitance of lipid bilayer.

System	Parameters	
	*R*_m_ [10^6^ Ω cm^2^]	*C*_m_ [10^−6^ μF/cm^2^]
DOPC	1.472 ± 0.07	0.617 ± 0.03
+1 mM/L CinA	2.419 ± 0.10	0.637 ± 0.06
+5 mM/L CinA	4.094 ± 0.05	0.682 ± 0.04
DOPC/PS 9:1	2.043 ± 0.04	0.628 ± 0.03
+1 mM/L CinA	3.111 ± 0.10	0.644 ± 0.05
+5 mM/L CinA	4.836 ± 0.05	0.675 ± 0.03
DOPC/PS 8:2	2.521 ± 0.07	0.640 ± 0.05
+1 mM/L CinA	3.941 ± 0.10	0.651 ± 0.03
+5 mM/L CinA	5.777 ± 0.06	0.678 ± 0.02

**Table 5:** Effect of *p*-coumaric acid on the resistance and capacitance of lipid bilayer.

System	Parameters	
	*R*_m_ [10^6^ Ω cm^2^]	*C*_m_ [10^−6^ μF/cm^2^]
DOPC	1.472 ± 0.07	0.617 ± 0.03
+1 mM/L *p*-CoA	2.267 ± 0.12	0.645 ± 0.05
+5 mmol/dm^3^ *p*-CoA	3.388 ± 0.08	0.743 ± 0.04
DOPC/PS 9:1	2.043 ± 0.04	0.628 ± 0.03
+1 mM/L *p*-CoA	2.877 ± 0.07	0.649 ± 0.02
+5 mM/L *p*-CoA	4.124 ± 0.04	0.733 ± 0.03
DOPC/PS 8:2	2.521 ± 0.07	0.640 ± 0.05
+1 mM/L *p*-CoA	3.505 ± 0.07	0.656 ± 0.04
+5 mM/L *p*-CoA	5.013 ± 0.04	0.743 ± 0.02

**Table 6:** Effect of ferulic acid on the resistance and capacitance of lipid bilayer.

System	Parameters	
	*R*_m_ [10^6^ Ω cm^2^]	*C*_m_ [10^−6^ μF/cm^2^]
DOPC	1.472 ± 0.07	0.617 ± 0.03
+1 mM/L FA	1.734 ± 0.04	0.651 ± 0.05
+5 mM/L FA	2.809 ± 0.08	0.755 ± 0.05
DOPC/PS 9:1	2.043 ± 0.04	0.628 ± 0.03
+1 mM/L FA	2.653 ± 0.07	0.651 ± 0.02
+5 mM/L FA	3.892 ± 0.04	0.743 ± 0.03
DOPC/PS 8:2	2.521 ± 0.07	0.640 ± 0.05
+1 mM/L FA	2.890 ± 0.07	0.657 ± 0.04
+5 mM/L FA	4.621 ± 0.04	0.748 ± 0.02

**Table 7:** Basic physicochemical parameters of cinnamic acid and its derivatives.

Compound	*M*_WT_^1^	*p*K_COOH_^2^	*p*K_OH_^3^	*c*log*P*^4^	log*D* ^5^	HBA ^6^	HBD ^7^
CinA	148.16 [18]	4.44 [53]	─	1.98 [54]	0.30 [18]	2 [55]	1 [55]
*p*-CoA	164.16 [18]	4.64 [56]	9.50 [56]	1.54 [54]	0.06 [18]	3 [55]	2 [55]
FA	194.18 [18]	4.46 [57]	8.75 [57]	1.42 [54]	−0.04 [18]	4 [55]	2 [55]

^1^ molecular weight, ^2^ dissociation constant of the carboxyl groups, ^3^ dissociation constant of the hydroxyl groups, ^4^ calculated n-octanol/water partition coefficient, ^5^ distribution coefficient at pH 5.2 ^6^ hydrogen bond acceptor groups, ^7^ bond donor groups.

## Abbreviations

ELS	Electrochemical light scattering
EIS	Electrochemical impedance spectroscopy
CinA	Cinnamic acid
*p*-CoA	*p*-Coumaric acid
FA	Ferulic acid
DOPC	1,2-Dioleoyl-sn-glycero-3-phosphocholine
PC	Phosphatidylcholine
PS	Phosphatidylserine
*C*_m_	Membrane capacitance
*R*_m_	Membrane resistance
σ	Surface charge density
log*P*	n-octanol/water partition coefficient
clogP	Calculated n-octanol/water partition coefficient
Ro5	Rule of five
*M*_WT_	Molecular weight
p*K*_a_	Dissociation constant
*p*K_COOH_	Dissociation constant of the carboxyl groups
*p*K_OH_	Dissociation constant of the hydroxyl groups
HBA	Hydrogen bond acceptor groups
HBD	Hydrogen bond donor groups
